# Uncontrolled donation after circulatory death: comparison of two kidney preservation protocols on graft outcomes

**DOI:** 10.1186/s12882-017-0805-1

**Published:** 2018-01-08

**Authors:** Claire Delsuc, Alexandre Faure, Julien Berthiller, Didier Dorez, Xavier Matillon, Vannary Meas-Yedid, Bernard Floccard, Guillaume Marcotte, Vanessa Labeye, Maud Rabeyrin, Ricardo Codas, Cécile Chauvet, Philip Robinson, Emmanuel Morelon, Lionel Badet, William Hanf, Thomas Rimmelé

**Affiliations:** 1 Hospices Civils de Lyon, Hôpital Edouard Herriot, Université Claude Bernard Lyon 1, service d’Anesthésie Réanimation, Lyon, France; 2 EA 7426 (Université Claude Bernard Lyon 1 – Hospices Civils de Lyon – bioMérieux) “Pathophysiology of Injury-Induced Immunosuppression – PI3”, Lyon, France; 3 Hospices Civils de Lyon, Hôpital Edouard Herriot, service d’Epidémiologie Clinique, Pôle Information Médicale Evaluation Recherche (unité statistiques), Lyon, France; 4 000 0004 0639 3167 Centre Hospitalier Annecy Genevois, service de Coordination des Prélèvements d’Organes et de Tissus, Metz-Tessy, France; 5 Hospices Civils de Lyon, Hôpital Edouard Herriot, service d’Urologie, Lyon, France; 6 CNRS UMR 3691, Institut Pasteur, Bioimage Analyse Unit, Paris, France; 7 Hospices Civils de Lyon, Hôpital Edouard Herriot, service de Coordination des Prélèvements d’Organes et de Tissus, Lyon, France; 8 Hospices Civils de Lyon, Hôpital Edouard Herriot, service d’Anatomopathologie, Lyon, France; 9 Hospices Civils de Lyon, Hôpital Edouard Herriot, Université Claude Bernard Lyon 1, service de Transplantation, Néphrologie et Immunologie Clinique, Lyon, France; 10 Hospices Civils de Lyon, Direction de la Recherche Clinique et de l’Innovation, Lyon, France; 11 Centre Hospitalier Alpes-Léman, service de Néphrologie, Contamine-sur-Arve, France; 12 Service d’Anesthésie Réanimation, Hôpital Edouard Herriot, Hospices Civils de Lyon, 5 place d’Arsonval, 69437 Lyon Cedex 03, France

**Keywords:** Kidney transplantation, Donor selection, Organ preservation, Graft survival, Glomerular filtration rate, Fibrosis

## Abstract

**Background:**

Kidney transplantation following uncontrolled donation after circulatory death (uDCD) presents a high risk of delayed graft function due to prolonged warm ischemia time. In order to minimise the effects of ischemia/reperfusion injury during warm ischemia, normothermic recirculation recently replaced in situ perfusion prior to implantation in several institutions. The aim of this study was to compare these preservation methods on kidney graft outcomes.

**Methods:**

The primary endpoint was the one-year measured graft filtration rate (mGFR). We collected retrospective data from 64 consecutive uDCD recipients transplanted over a seven-year period in a single centre.

**Results:**

Thirty-two grafts were preserved by in situ perfusion and 32 by normothermic recirculation. The mean ± SD mGFR at 1 year post-transplantation was 43.0 ± 12.8 mL/min/1.73 m^2^ in the in situ perfusion group and 53.2 ± 12.8 mL/min/1.73 m^2^ in the normothermic recirculation group (*p* = 0.01). Estimated GFR levels were significantly higher in the normothermic recirculation group at 12 months (*p* = 0.01) and 24 months (*p* = 0.03) of follow-up. We did not find any difference between groups regarding patient and graft survival, delayed graft function, graft rejection, or interstitial fibrosis.

**Conclusions:**

Function of grafts preserved by normothermic recirculation was better at 1 year and the results suggest that this persists at 2 years, although no difference was found in short-term outcomes. Despite the retrospective design, this study provides an additional argument in favour of normothermic recirculation.

**Electronic supplementary material:**

The online version of this article (10.1186/s12882-017-0805-1) contains supplementary material, which is available to authorized users.

## Background

To reduce organ shortage, several countries started donation after circulatory death (DCD) programs. According to some experts, the organs from DCD have the potential to significantly increase the pool of kidneys by up to 20–30% [1, 2]. However, using such donors may also adversely affect graft outcomes. Compared with donation after brain death, DCD kidneys experience higher rates of delayed graft function (DGF) due to warm ischemia lesions [3]. Nevertheless, the incidence of DGF does not seem to affect long-term graft survival in DCD kidneys [3–5]; some authors have reported better long-term graft outcome with kidneys from DCD as compared to brain dead donors with expanded criteria, and similar graft outcomes as compared to kidneys from brain dead donors with standard criteria [3, 6].

The Maastricht classification defined four DCD categories according to the circumstances of donor death (including controlled and uncontrolled donors) [7]. Many countries such as United Kingdom, Belgium, USA, and Australia successfully developed controlled DCD programs and reported promising results [8, 9]. Due to ethical concerns, the French program started in 2006 with uncontrolled DCD (uDCD) and a restriction to kidney and liver transplantations [10]. We recently reported the first results of this program that found absence of primary non-function (PNF) in the first 27 transplantations, most likely due to a careful graft selection and the use of pulsatile machine perfusion during the cold ischemic period [11].

In uDCD, a degree of warm ischemia time is unavoidable and there is always a period of time during which the family consent and the donor medical history are obtained [3, 12]. To preserve organs during this period, in situ perfusion (ISP) was developed in the seventies and consists of infusing a cold preservation solution through an intra-aortic triple lumen catheter [13, 14]. More recently, Spanish teams proposed the use of normothermic recirculation (NR), otherwise known as normothermic extracorporeal membrane oxygenation [15]. NR was initially described for liver transplantation and to be more effective than ISP to reduce the detrimental effects of warm ischemia [16]; preliminary studies reported similar benefits for renal transplantation in uDCD, NR decreasing the DGF rate as compared to ISP [17–19].

In 2006, the French national uDCD program started in our centre, using ISP as the kidney preservation protocol. After promising preliminary results reported in the literature, we switched to NR in January 2010 [18, 19]. To our knowledge, there is no published study that has investigated long-term graft outcome or histology improvement with the use of NR. Therefore, the aim of this study was to compare the graft outcomes from uDCD kidney recipients preserved either by ISP or NR.

## Methods

### Study design and patients

We conducted a retrospective study in an academic hospital where patients received kidney grafts from uDCD. All cases were consecutive recipients and we collected data on each uDCD recipient transplanted between September 2006 and September 2013 using medical records. We included 27 patients from a previous study, all preserved by ISP [11]. Kidneys were retrieved from two French hospitals: Edouard Herriot public teaching hospital in Lyon, and the public hospital of Annecy, France. We did not include recipients transplanted outside our centre with grafts from those 2 retrieving centres. The end of the follow-up period was October 2015; all patients were followed for 2 years.

uDCD inclusion criteria were based on the French uDCD program, as follows: out-of-hospital cardiac arrest (Maastricht I) or in-hospital cardiac arrest (Maastricht II), with a unsuccessful resuscitation, a precise time of cardiac arrest, a duration without cardiopulmonary resuscitation less than 30 min (no flow period), age ≥ 18 to ≤55 years and an interval before preservation protocol initiation <150 min [20]. Donors with history of chronic kidney disease, hypertension, diabetes, sepsis, neoplasm, intravenous drug addiction, or traumatic cardiac arrest were not included. We also did not include patients eligible for extracorporeal life support or prolonged cardiopulmonary resuscitation (no flow less than 5 min, capnography greater than 15 Torr (2 kPa) after 20 min of resuscitation, hypothermia, drug intoxication, or signs of life during cardiopulmonary resuscitation).

Recipient criteria were age ≤ 60 years, first kidney transplantation, ABO compatibility, no previous HLA-sensitisation, and agreement to potentially receive a graft from an uDCD.

### Organ preservation protocol

**Fig. 1:**
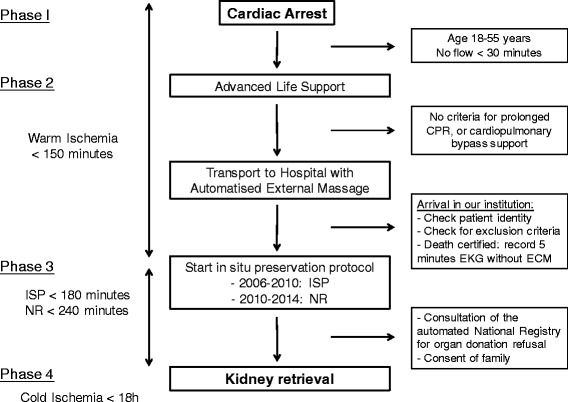
Protocol of care for uncontrolled donors after circulatory death in our retrieving centres. The different steps, timing, and exclusion criteria are described. No flow has to be less than 30 min, and advanced life support provided a least 30 min before considering uDCD. Time between cardiac arrest to efficiency preservation protocol has to be less than 150 min. Using NR preservation protocol allows 240 min before kidney retrieval, while ISP allows a 180 min interval. CPR: cardiopulmonary resuscitation, EKG: electrocardiogram, ECM: external cardiac massage, ISP: in situ perfusion, NR: normothermic recirculation

Patients with out-of-hospital cardiac arrest were handled on-site by the emergency medical services with advanced life support. If fulfilling the criteria for uDCD, patients were referred to the uDCD center of our institution, under mechanical ventilation and continuous external cardiac massage. Upon arrival, death was certified by a 5-min electrocardiogram confirming the absence of spontaneous cardiac activity. Standard blood tests were then performed as a conventional prerequisite for donation. The entire uDCD procedure of our institution is described in Fig. 1. From September 2006 to January 2010, after confirmation of death, the ISP preservation protocol was initiated. An intraaortic double-balloon catheter (Gillot catheter) and a venous vent were surgically inserted through the right side of the groin, with an injection of 25,000 IU of heparin. The intraaortic catheter was perfused with a heparinized (5000 IU/L) preservation solution at 4 °C (IGL-1, Institut Georges Lopez, Saint-Didier-au-Mont-d’Or, France) at a rate of 250 to 500 mL/min until blood washout, and maintained at 100 mL/min. Finally, peritoneal refrigeration with 4 L isotonic saline at 4 °C was performed. ISP duration had to be less than 180 min. From February 2010, we used cardiopulmonary bypass for organ procurement as follows: after cannulation of the femoral artery and vein, cannulas were connected to a blood oxygenator, a heat exchanger and a non-pulsatile roller pump. This NR was run at the minimum pump flow rate of 2 L/min, with an oxygen concentration set to 100%. NR duration had to be less than 240 min. The automated national registry for organ donation refusal was consulted (*registre national des refus*), and donor family consent was obtained for all uDCD kidneys.

Cold ischemia time after kidney retrieval was managed with a pulsatile hypothermic (1–4 °C) machine perfusion (RM3, Waters Medical Systems, Rochester, MN, USA, or LifePort, Organ Recovery System, Des Plaines, IL, USA) and the organ preservation solution was the Belzer MPS Solution (Waters Medical Systems) or KPS-1 (Organ Recovery System). Grafts were preserved by this machine perfusion until the end of the cold ischemia time. Organ viability was assessed by measuring ex vivo intrarenal vascular resistance index.

### Immunosuppression

Patients received antithymocyte globulin (Thymoglobulin, Genzyme, Cambridge, MA, USA) for induction therapy (1.25 mg/kg/day for 10 days). Maintenance immunosuppression included three types of drug. Calcineurin inhibitors were introduced on day 6–8; cyclosporine (Neoral, Novartis Pharma AG, Basel, Switzerland) or tacrolimus (Prograf, Astellas Pharma Inc., Tokyo, Japan) doses were adjusted to obtain trough levels between 100 and 150 ng/mL and 8–12 ng/mL, respectively, during the first year. Mycophenolate mofetil 2 g/day adapted to patient tolerance (Cellcept, Roche Pharmaceuticals, Basel, Switzerland). Steroids were quickly tapered to 5 mg/day within the first 2 months after transplantation. Cyclosporine was used for patients with body mass index >25 kg/m^2^ and/or past medical history of diabetes, and tacrolimus for the others. This protocol remained unchanged during the study period.

### Patient and graft outcomes

Graft and patient survival at 24 months (M24) were analysed, graft loss being defined by requirement of chronic dialysis. Short-term outcome was evaluated by PNF, DGF rates, duration of dialysis after transplantation, and number of days to obtain a urine output above 1000 mL. PNF was defined as an immediate and permanent non-function of the graft after transplantation requiring chronic dialysis (and chronic dialysis was defined by more than 3 months of dialysis). DGF was defined as the need for at least one dialysis session during the first week after transplantation, except for post-transplantation hyperkalaemia. Measured GFR (mGFR) was performed with inulin or iohexol clearance at M12. We assessed graft function by estimated GFR (eGFR) with simplified modification diet renal disease (MDRD) formula (at 1, 3, 6, 12, and 24 months after transplantation: M1, M3, M6, M12, and M24) [21]. Clinical acute rejection was defined as a serum creatinine increase of more than 20% over baseline. These acute rejections were biopsy-proven where possible. Subclinical rejection without creatinine increase was diagnosed with protocol biopsies at M3 and M12.

### Histological assessment

Protocol graft biopsies were performed at M3 and M12. Kidney core biopsies were screened by the same pathologist according to the Banff 07 classification [22]. Masson-stained biopsy slides were subsequently digitised to quantify interstitial fibrosis by automatic colour image analysis, as previously described in a robust and reproducible manner [23, 24].

### Statistical analysis

Categorical variables were expressed as number (*n*) and percentage, and quantitative variables were expressed as mean ± standard deviation. The hypothesis of normal distribution of quantitative variables was tested using the Kolmogorov-Smirnov test and graphically confirmed with a histogram. If necessary, the variable was converted to Log and/or specifically analysed after exclusion of outliers.

Categorical variables were compared using the Chi square test or Fisher’s exact test when the conditions of application of Chi square test were not met. Quantitative variables were compared between groups using Student’s t test after verification of equality of variances when data were normally distributed, and with the Wilcoxon nonparametric test when the hypothesis of normality of distribution was not verified.

Survival curves for graft and patient survival were obtained using the Kaplan-Meier method and compared using the log-rank test. Graft survival was determined between date of transplantation to date of chronic dialysis requirement. Patient overall survival was defined by date of death.

Comparison of mean mGFR values at 12 months between the two procedures were adjusted for recipient age and gender, cold ischemia time, and NR/ISP duration using a MANOVA (GLM SAS procedure).

The statistical level of significance was set to 5% (*p* < 0.05). Statistical analyses were conducted using SAS version 9.3 (SAS Institute Inc., Cary, NC, USA).

## Results

### Study population

A total of 64 renal transplant recipients from uDCD were included, all Maastricht I category. They were classified according to their kidney donor preservation group: ISP (*n* = 32) and NR (*n* = 32). Donor characteristics were not statistically different between the two groups. The number of HLA mismatches were more frequent in the NR group (mean ± SD = 4.8 ± 1.2 vs. 4.1 ± 1.1; *p* = 0.04), and duration of NR was longer than that of ISP (mean ± SD = 203 ± 46 min vs. 165 ± 44 min; *p* = 0.001), whereas duration of cold ischemia was longer in the ISP group (mean ± SD = 1027 ± 250 min vs. 817 ± 211 min; *p* = 0.001; Table 1).

**Table 1:** Characteristics of the Donors and Recipients

	ISP	NR	*p*
Donors	*n = 22*	*n = 24*	
Age, yr	41.8 ± 10.1	43.2 ± 8.6	0.61^a^
Male, *n* (%)	32 (100)	27 (84)	0.05^b^
Blood type, *n* (%)			0.10^b^
A	19 (59.4)	16 (50.0)	
B	5 (15.6)	1 (3.1)	
AB	0 (0.0)	2 (6.3)	
O	8 (25.0)	13 (40.6)	
Serum creatinine	144.1 ± 44	130.3 ± 36	0.32^a^
eGFR (MDRD)	55.1 ± 20.7	58.1 ± 19.7	0.57^a^
No flow duration, min	10 ± 5	10 ± 10	0.38^a^
Low flow duration, min	118 ± 14	123 ± 20	0.16^a^
Recipients	*n = 32*	*n = 32*	
Age, yr	45.8 ± 11.1	47.9 ± 10.7	0.52^a^
Male, *n* (%)	25 (78)	24 (75)	0.77^c^
Diabetes, *n* (%)	1 (3)	3 (9)	0.61^b^
Blood type*, n* (%)			0.26^b^
A	19 (59.4)	14 (43.8)	
B	4 (12.5)	2 (6.3)	
AB	1 (3.1)	4 (12.5)	
O	8 (25.0)	12 (37.5)	
Waiting time on list >950 days, *n* (%)	26 (83.9)	24 (75.0)	0.38^a^
Cold ischemia, min	1027 ± 250	817 ± 211	0.001^a^
NR or ISP duration, min	165 ± 44	203 ± 46	0.001^a^
ABDR mismatch	4.1 ± 1.1	4.8 ± 1.2	0.04^a^

Continuous data are expressed as mean ± standard deviation

*ISP* in situ perfusion, *NR* normothermic recirculation, *eGFR* estimated glomerular filtration rate

^a^Wilcoxon test

^b^Fisher exact test

^c^Chi square

### Patient and kidney graft survival

Overall survival at M24 was not statistically different (log-rank test: *p* = 0.14) between the ISP and NR groups, respectively 100 and 92.6% (one patient died at M8 from pneumonia, another one at M15 after severe trauma). Kidney graft survival at M24 was also not statistically different (log-rank test: *p* = 0.27) between the ISP (96.8%) and NR (96.5%).

### Early graft outcome

We observed one PNF in each group. The incidence of DGF was not statistically different between groups (ISP: *n* = 27, 84% and NR: *n* = 23, 72%; *p* = 0.23). There were five complications in the ISP group (one arterial stenosis, two ureteral stenoses, two venous thromboses) and six in the NR group (five arterial stenoses and one postoperative haemorrhage). The median [IQR] duration of hospital stay for patients receiving kidneys preserved by NR was significantly 1 week shorter than that of patients receiving kidneys preserved by ISP (17 [15–21] vs. 24 [18–33] days, *p* = 0.003; Table 2).

**Table 2:** Early Recipient Grafts Outcome

	ISP *n* = 32	NR *n* = 32	*p*
Primary non-function, *n* (%)	1 (3)	1 (3)	1^b^
Delayed graft function, *n* (%)	27 (84)	23 (72)	0.23^c^
Operative complication, *n* (%)	5 (16)	6 (19)	0.74^c^
Duration of hemodialysis, days [range]	15 [9–22]	8 [1–16]	0.05^a^
Time for diuresis >1000 mL, days [range]	5 [1–16]	2 [1–11]	0.26^a^
*Missing, n*	*3*	*1*	
Length of stay, days	24 [18–33]	17 [15–21]	0.003^a^

Continuous data are expressed as median [interquartile range]

*ISP* in situ perfusion, *NR* normothermic recirculation

^a^Wilcoxon test

^b^Fisher exact test

^c^Chi square

### Graft function during follow-up

**Fig. 2:**
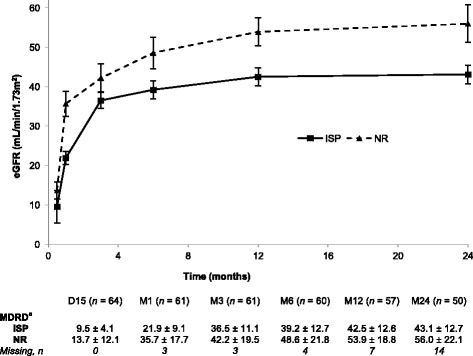
Evolution of estimated glomerular filtration rate over the first two years after transplantation. Comparative renal graft function (mean ± SD eGFR based on the simplified MDRD formula) in uDCD renal transplants preserved by ISP or NR. ^a^Estimated glomerular filtration rate in mL/min/1.73m^2^; mean ± SD. ISP: in situ perfusion, NR: normothermic recirculation

The one-year mean ± SD mGFR of the NR group (*n* = 24) was 53.8 ± 12.8 mL/min/1.73 m^2^ and that of the ISP group (*n* = 28) was 43.0 ± 12.8 mL/min/1.73 m^2^ (*p* = 0.007). The multivariate analysis confirmed this finding; the difference remained significant after adjusting for recipient age and gender, cold ischemia time and NR/ISP duration (*p =* 0.03), see Additional file 1. Mean eGFR was statistically different between groups at 1 year (*p* = 0.01) and 2 years (*p* = 0.03) favouring NR, but not at day 15 (*p* = 0.36; Figure 2).

### Histological assessment

The total number of adequate protocol biopsies was 34/64 at M3 and 32/64 at M12. These were not performed for 36% of patients at M3 and 42% of patients at M12 due to patient refusal, death, anticoagulant treatment, or post-transplantation arterio-venous fistula; other biopsies were not sufficient to perform histological assessment and were discarded.

The incidence of clinical acute rejection did not statistically differ between the ISP and the NR groups (19% vs. 18%; *p* = 0.93), but the incidence of borderline changes observed on the systematic biopsies was statistically more frequent in the ISP group than in the NR group (56% vs. 22%, *p* = 0.002). The mean interstitial fibrosis score was not different between groups at M3, or at M12. However, there was a trend towards lower interstitial fibrosis in the NR group at M12 (mean ± SD = 0.30 ± 0.10 vs. 0.37 ± 0.12). Concerning the Banff 07 score, the distribution of cv score at M3 and at M12 was not significantly different between the 2 groups (Table 3).

**Table 3:** Graft Rejection and Histological Assessment

	ISP *n* = 32	NR *n* = 32	*p*
M3 Fibrosis, *Mean ± SD*	0.31 ± 0.09	0.32 ± 0.14	0.68^d^
*Missing, n*	*14*	*16*	
M12 Fibrosis, Mean ± SD	0.37 ± 0.12	0.30 ± 0.10	0.08^a^
*Missing, n*	*16*	*16*	
Clinical acute rejection, n (%)	6 (19)	5 (18)	0.93^c^
*Missing, n*	*1*	*5*	
Borderline changes			
M3, n (%)	10 (45)	4 (21)	0.10^c^
*Missing, n*	*10*	*13*	
M12, n (%)	13 (68)	4 (22)	0.008^b^
*Missing, n*	*13*	*14*	
cv score M3, n (%)			0.83^b^
0	5 (25)	5 (33)	
1	11 (55)	6 (40)	
2	2 (10)	3 (20)	
3	2 (10)	1 (7)	
*Missing, n*	*12*	*17*	
cv score M12, n (%)			0.39^b^
0	5 (26)	8 (44)	
1	12 (63)	6 (33)	
2	1 (5)	2 (11)	
3	1 (5)	2(11)	
*Missing, n*	*13*	*14*	

*ISP* in situ perfusion, *NR* normothermic recirculation

^a^Wilcoxon test

^b^Fisher exact test

^c^Chi square

^d^Student test

## Discussion

In this study, the use of NR during uDCD procedure showed promising kidney graft outcome as assessed by renal function measurement and histological analysis. The NR uDCD kidney graft function (mGFR) was superior to the ISP group at 1 year and this difference remained significant after adjusting for cold ischemia time and preservation duration. As expected this difference was also found for eGFR at the same time point. In histological analysis, the trend found towards a greater proportion of fibrosis in the ISP group at 12 months was not found at 3 months; the significantly greater number of grafts with borderline changes at 12 months was also not found at 3 months. Similarly, there was no significant difference in most early outcome endpoints (such as PNF and DGF), and no difference in eGFR at day 15. This suggests that in the present study the benefit due to NR occurred later rather than in the immediate period of time following transplantation.

Our results should be seen in the context of machine perfusion after graft removal, which demonstrated efficiency in terms of reduction of DGF rate post-transplantation in DCD [25, 26]. This may have affected the results obtained in the entire cohort, but not the difference found between the two groups as machine perfusion was used in all grafts of this study. However, to increase the number of patients we included two different uDCD retrieving centres in our study. This led to a potential limitation as these two centres use different perfusion machine devices (RM3 and LifePort). But it is of note that there was no other difference in uDCD management as the two centres used exactly the same retrieving protocol. Both devices have been proven to be superior to cold storage in the literature [25–27], and the few studies comparing the two devices show no difference in graft function [28, 29], we therefore believe that any potential bias due to the machine is minimal.

A strength of the present study is that donor and recipient characteristics were not different between groups with regards to the duration without cardiopulmonary resuscitation (no flow) as well as the duration of cardiopulmonary resuscitation before preservation (low flow). Furthermore, both groups experienced the same operative complication rate, and postoperative immunosuppression protocols and management were identical. The duration of NR was, however, significantly longer than ISP but this was due to the institutional protocol (Fig. 1). More importantly, the mean duration of cold ischemia was more than 3 h longer in the ISP group, which is a potential confounder to properly compare the two techniques. Cold ischemia time is a well-known risk factor for graft and patient survival [30–32], however graft and patient survival were both high and no statistical difference between groups was found. In our study all grafts were preserved with machine perfusion. The optimal cold ischemia duration with a pulsatile hypothermic perfusion machine remains unclear. A recent study reported that prolonged cold ischemia with such devices was not associated with unfavourable one-year graft function outcome or graft survival [33]. Furthermore, in the comparisons of one-year mGFR, we adjusted for the impact of cold ischemia time and the difference still remained statistically significant. The retrospective design is also a limit as donor groups were not randomly assigned, yet the choice of intervention is unlikely to have been directed by patient characteristics as the preservation protocol was switched from ISP to NR between January and February 2010; this does not preclude, however, changes over time in other aspects of patient care.

We did not find any significant decrease in PNF or DGF between both groups. Previous studies in controlled and uncontrolled DCD cohorts found different results [18, 34–37]. In 48 uDCD recipients (40 with ISP, and 8 with NR), Valero et al. found an important and significant reduction of DGF rate using NR (from 55 to 12.5%), as did Demiselle et al. (from 81 to 53% in 50 uDCD recipients; 31 with ISP and 19 with NR); however, no data were published regarding long-term outcomes [18, 36]. Interestingly, our study found a higher DGF rate than that reported by Valero et al. [18] and which was in the upper range of values reported in the literature (12.5–92%) [10, 19, 34, 36]. However, we found a shorter length of stay duration in the NR, probably due to fewer dialysis sessions. DGF is assumed to be deleterious in recipients of brain dead donors for long-term kidney graft outcomes. In DCD recipients, DGF does not seem to influence kidney graft outcomes [35, 38]. The French uDCD program showed interesting results in terms of outcome and graft function, similar to extended criteria donors [11, 36]. Moreover, some studies reported favourable outcomes in kidneys transplanted from controlled DCD donors, comparable to outcomes of kidneys transplanted from donors after brain death [5, 6, 35, 38].

While the incidence of acute clinical rejection did not differ between the two groups, we observed a higher proportion of borderline changes in the ISP group at M12 despite them having a lower degree of HLA mismatch compared to the NR group. However, due to the retrospective design of the study, baseline biopsies were not available, but this difference of borderline changes was not found at M3, and appeared at M12. It has been suggested that NR could act as an ischemic preconditioning and may blunt the ischemia-reperfusion injury [16]. Indeed, by restarting circulation after cardiac arrest, the ischemic events surrounding circulatory arrest can be turned into an ischemic preconditioning phenomenon [39, 40]. In the recent study reported by Viglietti et al., the authors found an early and increased post-transplantation fibrosis in kidneys procured from uDCD compared to brain dead donors, which was associated with the duration of the no flow period (cut-off of 10 min) and explained a lower eGFR at 1 year post transplantation in the uDCD group [41]. We also investigated interstitial fibrosis in the present study, and it is interesting to highlight the quantitative analysis that we used which is relatively novel, and which we have already used to compare recipients of uDCD to brain dead donor grafts [11]. The small number of protocol biopsies is a limit of the study, but the rate of these was similar in the two groups and we were able to analyse histology in approximately half of the patients in each group. We observed a trend towards more frequent lower 12-month interstitial fibrosis score in the NR as compared to the ISP group. This suggests a certain potential effectiveness in this regard for this preservation protocol in uDCD recipients.

## Conclusion

In conclusion, our study found better results for NR preservation in uDCD as compared to ISP preservation in terms of graft function and borderline changes. However, we did not find any difference in short-term outcomes. This is the first study comparing these two methods on long-term graft outcome in uDCD. The retrospective design is a limit that prevents to definitely conclude to NR as the method of choice for uDCD kidneys procurement, but provides an additional argument in favour of NR.

## Additional file

Additional file 1: Multivariate analysis for one-year mGFR. Comparison between NR and ISP groups with MANOVA analysis adjusting for age, gender, cold ischemia time and NR/ISP duration. (DOCX 48 kb)

## Abbreviations

DCD: Donor after circulatory death
DGF: Delayed graft function
eGFR: Estimated glomerular filtration rate
ISP: In situ preservation
MDRD: Modification diet renal disease formula
mGFR: Measured glomerular filtration rate
NECMO: Normothermic extracorporeal membrane oxygenation
NR: Normothermic recirculation
PNF: Primary non-function
uDCD: Uncontrolled donor after circulatory death
