# Supplementary material for: Uncontrolled donation after circulatory death: comparison of two kidney preservation protocols on graft outcomes
Source: BMC Nephrol. 2018 Jan 8;19:3. doi: 10.1186/s12882-017-0805-1 (PMC5759186; doi:10.1186/s12882-017-0805-1)
Supplement: Additional file 1: — Multivariate analysis for one-year mGFR. Comparison between NR and ISP groups with MANOVA analysis adjusting for age, gender, cold ischemia time and NR/ISP duration. (DOCX 48 kb) [file 12882_2017_805_MOESM1_ESM.docx]

**Additional file 1. Multivariate analysis for one-year mGFR.** Comparison between NR and ISP groups with MANOVA analysis adjusting for recipient age and gender, cold ischemia time and NR/ISP duration.

|  | **Mean square** | **F value** | ***p*** |
| --- | --- | --- | --- |
| Age recipient | 129.8 | 0.7 | 0.39 |
| Gender recipient | 16.5 | 0.09 | 0.76 |
| Cold ischemia time | 207.2 | 1.2 | 0.28 |
| NR/ISP duration | 279.7 | 1.6 | 0.21 |
| NR/ISP | 918.9 | 5.2 | 0.03 |

mGFR: measured glomerular filtration rate, ISP: *in situ* perfusion, NR: normothermic recirculation.
